# Improvements of right ventricular function after intervention with CPAP in patients with obstructive sleep apnoea

**DOI:** 10.1186/s44156-024-00058-9

**Published:** 2024-10-01

**Authors:** Greg Murphy, Peter Coss, Gerard King, Mark Coyle, Anne-Marie McLaughlin, Ross Murphy

**Affiliations:** 1 St James’ Hospital, St James street, Dublin 8, Republic of Ireland; 2 Caardiology, Eagle Lodge, Limrick, Ireland; 3 Trinity College Dublin, Trinity College Dublin, Dublin, Ireland

**Keywords:** Obstructive sleep apnoea, Right heart, Echocardiography, CPAP

## Abstract

**Background:**

Obstructive sleep apnoea (OSA) is present in 40–80% of patients with cardiovascular morbidity and is associated with adverse effects on cardiovascular health. Continuous positive airway pressure (CPAP) maintains airway patency during sleep and is hypothesised to improve cardiac function. In the present study, we report on the impact of 12 weeks of CPAP and improvements in echocardiographic parameters of the right ventricle (RV).

**Methods:**

Nineteen newly diagnosed patients with OSA and a respiratory disturbance index (RDI) greater than 10 were enrolled. Echocardiography was performed before treatment and with a follow-up assessment after 12 weeks of CPAP. Echocardiographic and Doppler measurements were made following the American Society for Echocardiography guidelines. The primary outcome was isovolumetric acceleration (IVA). Secondary outcomes include tricuspid annular plane systolic excursion (TAPSE), fractional area change (FAC), RV % strain, TEI index and RV dimension (RVD1).

**Results:**

There was significant improvement in isovolumetric acceleration of 0.5ms^2^ (*P* = 0.0012 (95% CI -0.72, -0.20)) and significant improvement of 2.05 mm in TAPSE (*p* = 0.0379 (95% CI -3.98 - -0.13). There was no significant difference in FAC, RV % strain, TEI index or RVD1 with twelve weeks of CPAP therapy.

**Conclusion:**

The present study highlights significant improvement in TAPSE and IVA with 12 weeks of CPAP treatment and no significant improvement in FAC, RVD1 and RV % strain. These data indicate favourable characteristics on both load dependent and load independent markers of RV function with CPAP.

## Background

Obstructive sleep apnoea (OSA) is a common condition characterised by intermittent airway obstruction and apnoeic episodes during sleep. This results in fragmented nonrestorative sleep and significantly impacts cardiovascular health [1]. The frequency among the general population is estimated at 2–7% and is underdiagnosed in the clinical setting. It has been reported to be as high as 40–80% in those with a cardiovascular comorbidity [2, 3].

There are multiple physiological mechanisms through which OSA is associated with adverse cardiovascular health outcomes. Firstly, arousal from sleep activates the sympathetic nervous system, increasing blood pressure and heart rate [4]. Secondly, increased intrathoracic pressure increases afterload [5]. Thirdly, hypoxemia induces tachycardia and may trigger dysrhythmias and demand-supply mismatch [5, 6]. By these mechanisms, OSA has been proven to be strongly associated with cardiovascular comorbidities, including atrial fibrillation, heart failure, pulmonary hypertension and myocardial infarction [7, 8]. Furthermore, Marin et al. demonstrated that untreated severe OSA significantly increased cardiac mortality [9].

The right heart acts as a conduit for the lungs and OSA has been associated with altered right heart physiology on echocardiography [10]. However, right ventricular (RV) function remains a challenge to assess given the position in the chest, complex anatomy and the interplay of contractility and load states. Due to these complexities, the best marker for RV function in patients with OSA remains elusive. One meta-analysis of 82 articles concluded a statistically significant negative impact on myocardial performance index (MPI), tricuspid annular plane excursion (TAPSE), and FAC in patients who have OSA but did not collect data on isovolumetric acceleration time (IVA) or on the impact of intervention with CPAP [11].

In the present study, we present the echocardiographic and Doppler features of intervention with continuous positive airway pressure (CPAP) on the mechanics and function of the right heart. The primary outcome is isovolumetric acceleration time (IVA). Secondary outcomes include TAPSE, RV per cent strain, Fractional area change (FAC) and RV dimension one (RVd1).

## Methods

### Study design

Nineteen newly diagnosed patients with OSA and a respiratory disturbance index (RDI) greater than 10 were enrolled from St James’ hospital sleep clinic. All patients consented to receive CPAP therapy and to partake in the study. A CPAP user was by convention accepted to be compliance/adherent if they achieve ≥ 4 h of use on 70% of nights, this was achieved on all our patients.

Echocardiography was performed before treatment and with a follow-up assessment after 12 weeks of CPAP. Conventional echocardiographic and Doppler measurements were made following the American Society for Echocardiography guidelines. Patient characteristics can be seen in Table 1.

At the echocardiographic examination, the subject’s height, weight, heart rate and blood pressure were recorded. The local hospital ethics committee approved this study, and informed consent was obtained from all participants.

All echocardiographic recordings were obtained in digital format and stored for offline analysis. The offline analysis of images was performed using commercially available software “Echo Pac V202 only” 2017–2018 Vingmed Ultrasound on a personal computer workstation.

### Outcomes

The primary outcome was isovolumetric acceleration (IVA). Myocardial acceleration during the isovolumic phase of contraction (IVA) is a relatively load-independent marker. IVA was recorded using a 2 mm pulsed‐wave TDI sample volume placed at the annulus and in basal and mid‐ventricular segments in the apical four‐chamber view and averaged. RV myocardial velocities during peak systole, early and late diastole were recorded and calculated (difference between baseline and peak myocardial systolic velocities divided by the time interval from onset of the myocardial velocity during isovolumic contraction to the time at peak velocity of this wave). The potential effect of respiration was minimised by averaging multiple consecutive beats. Means of measuring IVA and its validity has been previously described [12–14]. 

Secondary outcomes included:

1. Tricuspid annular plane systolic excursion (TAPSE),
2. Right ventricle dimension size at the tricuspid annulus (RVD1),
3. TEI index,
4. fractional area change (FAC),
5. right ventricle per cent strain (RV %),

#### TAPSE

TAPSE was obtained by placing the M-mode line at the lateral tricuspid valve annulus, obtaining an M-mode tracing and measuring the height of the annulus movement during systole.

#### TEI index

calculated as isovolumic relaxation time plus isovolumic contraction time divided by ejection time. TEI index was not recorded in one post-CPAP participant due to image quality.

#### Fractional area change

Fractional area change was calculated in the apical four-chamber view as the difference in the end-diastolic area and the end-systolic area divided by the end-diastolic area.

#### Right ventricle per cent strain

RV per cent strain was performed on the apical four-chamber view. We evaluated the average value of the peak systolic strain from all segments of the free wall and septal wall of the RV in the apical four-chamber view, focused on the RV. Two data entries were not possible in pre-CPAP and two in post CPAP groups due to limited image quality of participants.

### Quality

Studies were completed by a qualified cardiac physiologist and stored offline. The two-dimensional longitudinal strain was measured using 2D deformation imaging (i.e. The per cent systolic deformation relative to the diastolic value) analysis software. Activation of the automatic ROI tracking mode ensured that measurements reflected the motion of myocardial tissue segments throughout the cardiac cycle. Images were acquired at a variable frame rate (50–70 s‾^10^) and stored digitally on a hard disc for offline analysis by speckle methodology. Not all outcomes could be measured in all studies due to patient factors and image quality.

### Statistical analysis

Data was blinded after collection and analysed in Microsoft Excel version 16.6. Mean standard deviations were collected. Statistical analysis was performed using the student’s T-test with an alpha of 0.05 using SPSS version 27.

## Results

### Patient characteristics

Patient characteristics can be seen in Table 1. Nineteen patients consented to partake in the study. There was a male gender majority (*N* = 17) with a mean BMI in the obese range. Other risk factors are seen in Table 1 below.

**Table 1:** Characteristics of participants

N = 19	
Age	52 yrs. (8)
Male gender	70% (N = 14)
Current smoker	14%
Ex-smoker	31%
Hypertension	46%
Systolic BP	129.3 (20.1)
Diastolic BP	88(12.6)
ODI (pre)	25(14)
ODI (Post)	4.2(2)
Epworth score (Pre)	25(14)
Epworth score (post)	7(4)
Neck Circumference	40 cm (2.1)
Ejection fraction	65% (8)
BMI	32(6)

(SD) = standard deviation

ODI = Oxygen desaturation index

BMI = Body mass index

### Isovolumetric acceleration

**Fig. 1:**
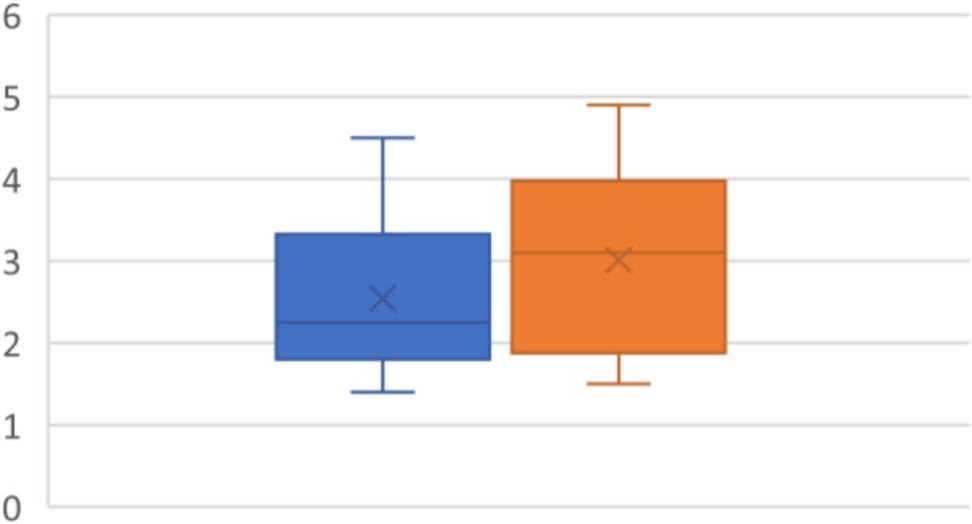
Isovolumetric acceleration (MS^2^) with pre-CPAP (blue) and post CPAP (Orange)

IVA was reported on all included patients in the study (*N* = 19). On diagnosis, the mean was 2.5+/- 1 MS^2^. After 12 weeks of CPAP, the mean was 3 +/- 1.1 MS^2^*P* < 0.0012 (95% CI -0.72 - -0.20MS^2^). There was a significant improvement in IVA with the adaption of CPAP (Fig. 1).

**Fig. 2:**
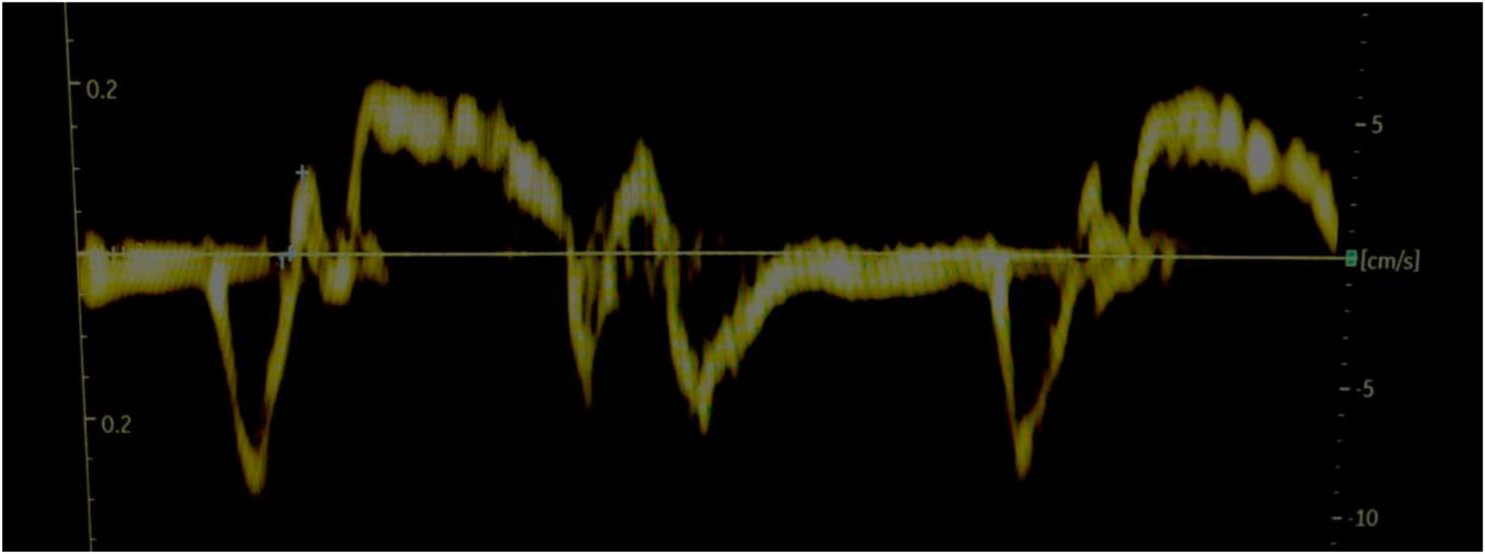
IVA measurement in patient A pre-CPAP demonstrating an isovolumetric acceleration time of 1.3 ms^2^

**Fig. 3:**
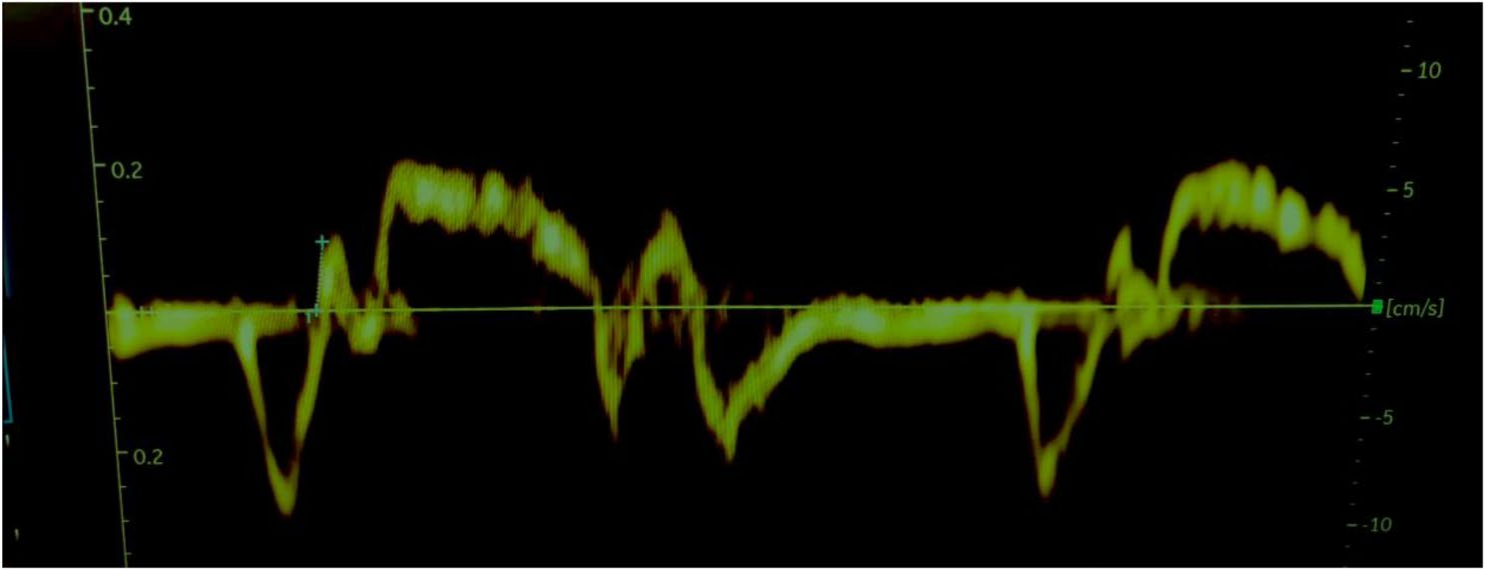
IVA measurement in patient A post-CPAP demonstrating an isovolumetric acceleration time of 2.1 ms^2^

### TAPSE

**Fig. 4:**
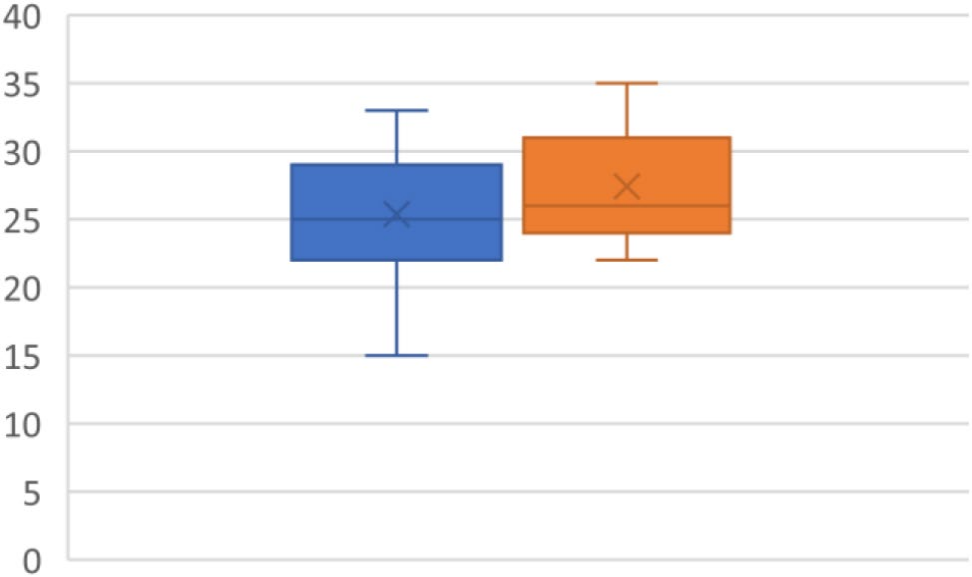
TAPSE in mm with pre-CPAP (blue) and post-CPAP (Orange)

TAPSE was reported for all patients in the study (*N* = 19). The mean on diagnosis was 25.37 mm(4.42) and 27.42 mm (3.91) after the intervention. P value was 0.038 (95% CI -3.98 mm - -0.13 mm), yielding a statistically significant result (Fig. 4).

### RVD1

**Fig. 5:**
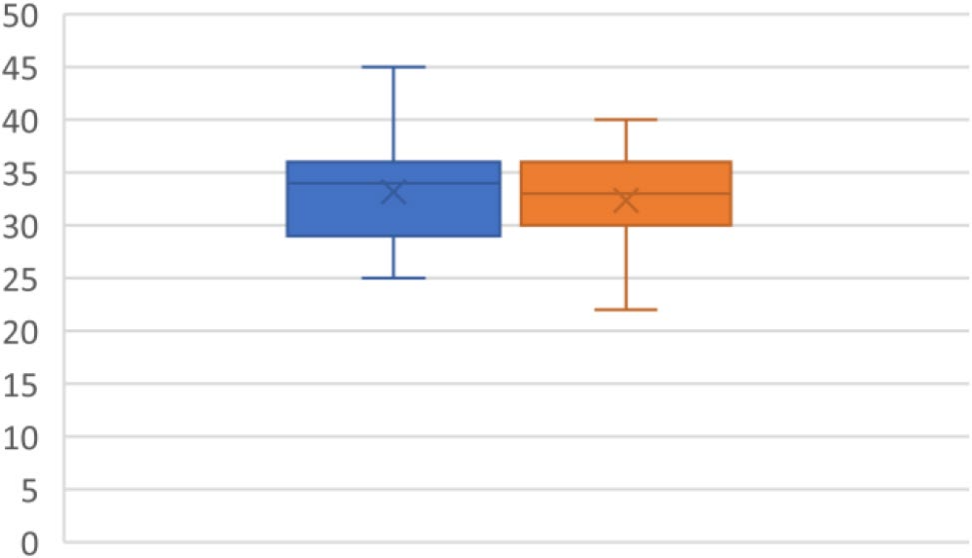
RVD1 in mm with pre-CPAP (blue) and post-CPAP (Orange)

RVD1 was recorded for all patients in the study. Before, CPAP measurement was 33.16 mm SD 4.74, and the post-intervention measurement was 32.37 mm. The difference was non-significant (*P* = 0.3986 (95% CI -1.13–2.71)) (Fig. 5).

### FAC

**Fig. 6:**
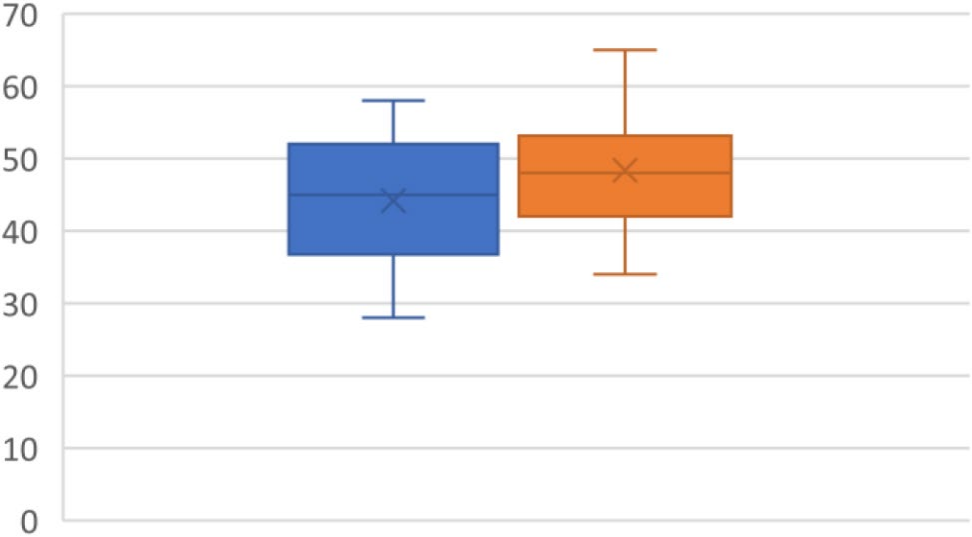
Fractional area change (%) with pre-CPAP (blue) and post-CPAP (Orange)

Fractional area change was analysed in 18 patients, with 44.17% (9.17%) and 48.39% (7.45%) post. The improvement was non-significant (*P* = 0.1109 (95% CI -9.52 -1.08)) (Fig. 6).

### TEI index

**Fig. 7:**
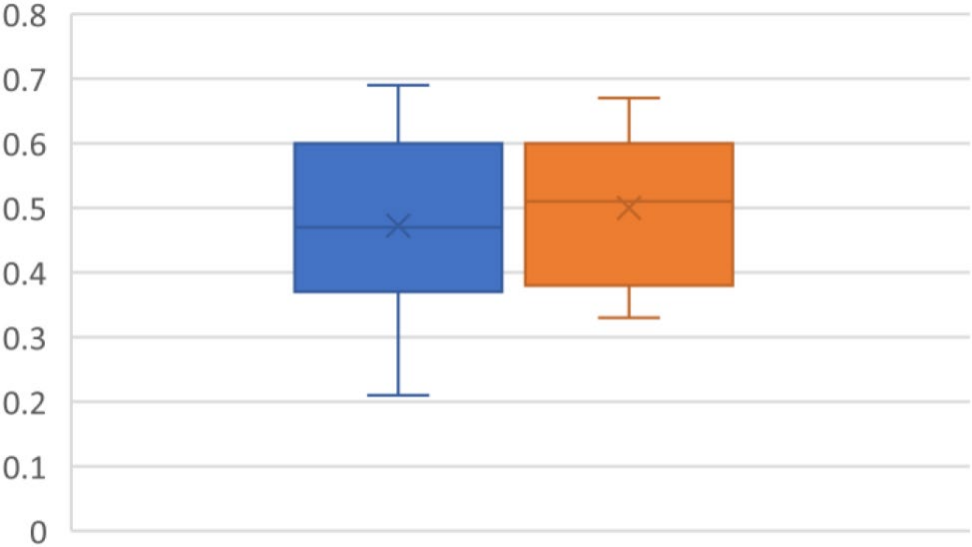
TEI index with pre-CPAP (blue) and post-CPAP (Orange)

The Tei index was 0.46 (0.13) pre and 0.5 (0.11) post-intervention. Indicating no significant difference. TEI (*P* = 0.4527 95% CI -0.11 - -0.05)(Fig. 7).

### RV % strain

**Fig. 8:**
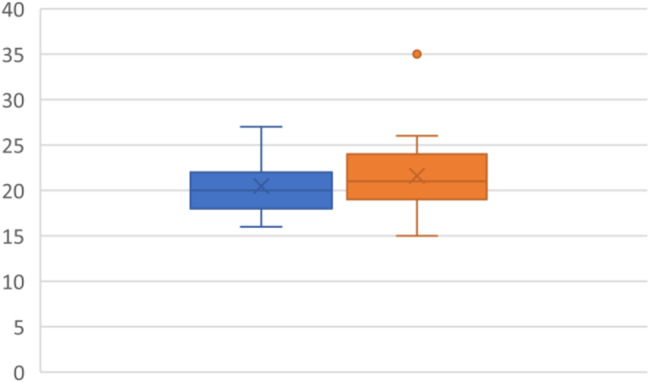
RV % strain with pre-CPAP (blue) and post-CPAP (Orange)

RV per cent strain was analysed in 15 patients. Mean pre intervention strain was 20.56 (3.58), post intervention mean 21.11(4.87). The RV per cent strain improvement did not reach significance (P=0.7370 (95% CI-3.99–2.88)) (Fig. 8).

**Table 2:** Interval change in RV function with the use of CPAP in patients with OSA

	On Diagnosis	After 12 weeks	P-Value
IVA N = 19	2.5 ± 1 ms^2^	3.0 +/- 1.1 ms^2^	< 0.0012 (95% CI -0.72 - − 0.20)
TAPSE N = 19	25.37 mm(4.42)	27.42 mm (3.91)	P = 0.0379 (95% CI -3.98– -0.13)
RV % strain N = 15	20.56%(3.58)	21.11%(4.87)	P = 0.7370 (95% CI-3.99–2.88)
Fractional area change N = 18	44.17%(9.17)	48.39%(7.45)	P = 0.1109 (95% CI -9.52–1–08)
RVD1 N = 19	33.16 mm(4.74)	32.37 mm(4.61)	P = 0.3986 (95% CI -1.13–2.71)
Tei index N = 18	0.46 (0.13)	0.50 (0.11)	P = 0.4001(95% CI -0.11. – 0.05)

*Paired T-test

## Discussion

The present study highlights subclinical changes in right ventricular mechanics with the application of CPAP therapy in OSA. There was a significant improvement in IVA and TAPSE with twelve weeks of treatment. There was no significant change in RV % strain, FAC, TEI index and RVD1 (Table 2).

IVA is a sensitive measure of contractility as a load independent marker of right heart function [13, 15, 16]. Arias et al. studied the isovolumetric phase of the left ventricle and demonstrated an improved isovolumetric relaxation time when patients were initiated on CPAP; however they did not report on IVA. They concluded that pressure overload from OSA impacts myocardial relaxation, and by offloading the ventricle with CPAP, myocardial mechanics can improve [17]. The isovolumetric phase has also been studied in other cardiac conditions. Ernande et al. showed that isovolumic contraction peak velocity at the tricuspid annulus > 9 cm/sec was an independent marker for death in pulmonary hypertension patients [18]. Furthermore, isovolumetric contraction time is an independent subclinical risk factor for heart failure in the general population [19]. To our knowledge, this is the first study of right ventricular IVA in an OSA cohort.

Conversely, TAPSE is a highly load dependent marker of RV systolic function and demonstrates a significant improvement with 12 weeks of CPAP (*P* < 0.05). Reduced TAPSE is associated with adverse outcomes in patients with heart failure and is an independent marker of mortality in aortic stenosis [20, 21]. We hypothesise that the significant improvement is likely due to the reduced afterload of CPAP therapy.

Secondary outcomes, including fractional area change, RVD1 and RV per cent strain did not demonstrate a significant improvement in function. TEI index, a marker of both systolic and diastolic function and an independent marker of mortality, did not show significant improvement with 12 weeks of therapy [22].

The data on RV mechanics with OSA is modest compared to studies of the left ventricle. A meta-analysis by Lu et al. demonstrated OSA to confer a significant reduction in TAPSE, MPI and RV FAC. The Wisconsin sleep study followed patients with OSA for a mean duration of 18 years and did not report a significant change in FAC in patients with OSA [23]. Both of these studies reported on the natural history of sleep apnoea and did not report on the impact of intervention with CPAP. Karamanzias et al. studied echocardiograms after one year of CPAP and found a significant improvement in TAPSE and no significant difference in RV diameter, which is consistent with our findings [24].

Speckle tracking and strain imaging allows for subclinical myocardial dysfunction to be identified. In contrast to our findings, Tadic et al. performed a meta-analysis of 337 patients and found a significant improvement in RV global longitudinal strain (GLS) with CPAP treatment (0.28 ± 0.07, CI 0.15–0.42, *p* < 0.0001), highlighting subclinical improvement in function [25].

The present study demonstrates significant improvements in TAPSE and IVA, highlighting improved myocardial mechanics across both load dependent and load independent markers of RV function when CPAP is used. These findings support greater screening and use of CPAP in this cohort; however; further data is needed on RV echo parameters in patients on CPAP.

### Limitations

A number of limitations should be addressed. Firstly the total number of included patients is modest. Secondly, while the studies were performed by an experienced sonographer interobserver variability and interpatient variability may limit wider application [26, 27]. Thirdly, some RV parameters could not be collected due to patient size and difficulty imaging the right ventricle. Fourthly, the sonographer was not blinded to which arm the patient was on at the time of the study. Finally, CPAP treatment is contingent on patient adherence, and low uptake has been well described in the literature [28].

## Conclusion

The present study highlights a significant improvement in TAPSE and IVA with CPAP treatment without a significant improvement in FAC, TEI index, RVD1 and RV % strain. These data indicate favourable characteristics with treatment and adherence to CPAP; however, more data is needed on right heart echocardiographic and Doppler mechanics and the clinical implications of different RV function markers.

## Data Availability

The data that support the findings of this study are not openly available due to reasons of sensitivity and are available from the corresponding author upon reasonable request. Data are located in controlled access data storage at St James Hospital.
